# Evaluation of repetitive-PCR and matrix-assisted laser desorption ionization-time of flight mass spectrometry (MALDI-TOF MS) for rapid strain typing of *Bacillus coagulans*

**DOI:** 10.1371/journal.pone.0186327

**Published:** 2017-10-11

**Authors:** Jun Sato, Motokazu Nakayama, Ayumi Tomita, Takumi Sonoda, Motomitsu Hasumi, Takahisa Miyamoto

**Affiliations:** 1 Safety Science Research, R&D, Kao Corporation, Ichikai, Tochigi, Japan; 2 Graduate School of Bioresource and Bioenvironmental Sciences, Kyushu University, Hakozaki, Higashi-ku, Fukuoka, Japan; 3 Health Care Food Research, R&D, Kao Corporation, Sumida, Tokyo, Japan; 4 Division of Food Science & Biotechnology, Department of Bioscience and Biotechnology, Faculty of Agriculture, Kyushu University, Hakozaki, Higashi-ku, Fukuoka, Japan; Bharathidasan University, INDIA

## Abstract

In order to establish rapid and accurate typing method for *Bacillus coagulans* strains which is important for controlling in some canned foods and tea-based beverages manufacturing because of the high-heat resistance of the spores and high tolerance of the vegetative cells to catechins and chemicals, matrix-assisted laser desorption/ionization time-of-flight mass spectrometry (MALDI-TOF MS) and repetitive-PCR (rep-PCR) were evaluated. For this purpose, 28 strains of *B*. *coagulans* obtained from various culture collections were tested. DNA sequence analyses of the genes encoding 16S rRNA and DNA gyrase classified the test strains into two and three groups, respectively, regardless of their phenotypes. Both MALDI-TOF MS and rep-PCR methods classified the test strains in great detail. Strains classified in each group showed similar phenotypes, such as carbohydrate utilization determined using API 50CH. In particular, the respective two pairs of strains which showed the same metabolic characteristic were classified into the same group by both MALDI-TOF MS and rep-PCR methods separating from the other strains. On the other hand, the other strains which have the different profiles of carbohydrate utilization were separated into different groups by these methods. These results suggested that the combination of MALDI-TOF MS and rep-PCR analyses was advantageous for the rapid and detailed typing of bacterial strains in respect to both phenotype and genotype.

## Introduction

Rapid identification of bacterial species and typing of strains are important for the assessment of clonal relationships among environmental isolates in food and beverage processing industries, especially when bacterial spoilage occurred on their products. The conventional methods of bacterial analysis, however, require high expertise, great experience, and long time for precise identification and typing. As a result of the development of the polymerase chain reaction (PCR) method in the 1990’s, molecular biological methods for identification of prokaryotes were established on the basis of the nucleotide sequences of 16S ribosomal RNA gene [1]. This bacterial identification technique spread widely in order to improve the bacterial control in food and beverage manufacturing. Evaluation of bacterial species could now be achieved within a day using this technique. Rapid typing methods of bacterial strains are needed to identify the origin of the spoilage bacteria as well as the identification of bacterial species. As nucleic acid-based typing methods, multilocus sequence typing (MLST) [2], pulse-field gel electrophoresis (PFGE) [3], random amplified polymorphic DNA (RAPD) [4], and Ribotyping [5, 6], are well known. Although MLST and PFGE have sufficient discrimination, these methods are time-consuming and labor intensive, requiring 2 working days for sample preparation and analysis. The RAPD method is capable of obtaining the results of strain typing within half a day. However, the reproducibility of band patterns obtained by amplification by arbitrarily primed PCR using short-length primers needs to be confirmed [7, 8]. In recent years, an automated microbial typing system with repetitive sequence-based PCR (rep-PCR) [9–11] and matrix-assisted laser desorption ionization time-of-flight mass spectrometry (MALDI-TOF MS) [12, 13] have been used for identification of bacterial species and may also provide potential alternative methods for strain typing. The genus *Bacillus* is also important target of strain typing for both clinical and industrial fields because of including pathogenic or food spoilage bacteria species such as *Bacillus anthrasis*, *B*. *cereus*, *B*. *licheniformis*, and *B*. *subtilis* [14, 15]. MALDI-TOF fingerprint and rep-PCR had potential to be obtained valuable information at both intra- and interspecies levels in the *Bacillus* species in previous study [14, 16]. Especially we focused on *Bacillus coagulans* which is well-known as a causative agent of flat-sour spoilage in some canned foods and tea-based beverages manufacturing [17, 18] as well as a representative bacterium for probiotics by lactic acid production [19]. In this study, we evaluated the reliability of rep-PCR and MALDI-TOF MS as strain typing of *Bacillus coagulans* by the comparison of ribotyping and phenotype of carbohydrate utilization.

## Materials and methods

### Bacterial strains and culture

Twenty-eight strains of *B*. *coagulans* were obtained from the National Institute of Technology and Evaluation (NITE), Japan Canner Association (JCA), and Deutsche Sammlung von Mikroorganismen und Zellkulturen GmbH (DSMZ) (Table 1). The type strain of *B*. *coagulans* NBRC12583^T^ was purchased from the NITE Biological Resource Center. Eighteen JCA strains were the isolates from various spoiled food products.

**Table 1:** *Bacillus coagulans* strains used in this study.

No.	Strain No.	Source
1	*^1^NBRC12583^T^	Evaporated milk
2	*^2^JCA1108	Spoiled canned mushrooms in spring water
3	JCA1109	
4	JCA1111	Canned curry
5	JCA1115	Canned crab meat
6	JCA1116	
7	JCA1117	Canned squid
8	JCA1118	Curry before canned
9	JCA1120	Canned sausage
10	JCA1121	
11	JCA1122	Canned flavored squid
12	JCA1131	Canned tuna
13	JCA1158	Canned mackerel in oil
14	JCA1174	Canned green peas in spring water
15	JCA1175	
16	JCA1177	Canned flavored arch clams
17	JCA1178	
18	JCA1180	Spoiled retort pouch food
19	JCA1182	Spoiled retort pouch sauce
20	*^3^DSM2308	-
21	DSM2311	-
22	DSM2312	-
23	DSM2314	Rhizosphere
24	DSM2350	-
25	DSM2356	-
26	DSM2383	-
27	DSM2384	-
28	DSM2385	-

-: Unknown

*1 NITE Biological Resource Centre

*2 Japan Canner Association

*3 Deutsche Sammlung von Mikroorganismen und Zellkulturen GmbH

*B*. *coagulans* strains were cultured on soybean-casein digested agar (Wako, Osaka, Japan) at 45°C for 2 days, and the absence of spores was confirmed by phase contrast microscopy.

### Matrix-assisted laser desorption ionization time-of flight mass spectrometry

Single colony was picked up from an agar plate and suspended in 300 μL distilled water. The cell suspension was mixed with 900 μL ethanol (Wako Pure Chemicals Inc., Osaka, Japan), and centrifuged at 13,000 rpm for 2 min at room temperature. The supernatant was discarded and residual ethanol was allowed to evaporate completely. Pellets were resuspended in 30 μL of 70% formic acid (Wako, Osaka, Japan), mixed with 30 μL acetonitrile (Wako Pure Chemicals Inc., Osaka, Japan) and centrifuged at 13,000 rpm for 2 min at room temperature. After a centrifugation, 1 μL of the supernatant was spotted onto a disposable steel plate (SYSMEX bioMérieux, Tokyo, Japan) and air dried at room temperature. Each sample was spotted three replicates on the plates. Sample spots were overlaid with 1 μL of matrix solution (α-cyano-4-hydroxy cinnamic acid; SYSMEX bioMérieux, Tokyo, Japan) and air dried at room temperature. The mass spectra were obtained using an AXIMA performance MALDI-TOF MS (Shimadzu Corporation, Kyoto, Japan) with a nitrogen laser (λ = 337.1 nm) and a mass range *m/z* from 3000 to 20,000. Shimadzu Biotech Launchpad software (Shimadzu Corporation, Kyoto, Japan) was used for mass spectra acquisition and peak detection. The calibration of mass spectra was performed using *Escherichia coli* every analysis. Cluster analysis of mass spectra was performed with SARAMIS software (database V4.10, Vitek MS plus; SYSMEX bioMérieux, Tokyo, Japan) to discriminate *B*. *coagulans* strains. A dendrogram was made with single link agglomerative clustering algorithm based on mass spectra. The strains having the mass spectra with more than 80% in similarity were defined as the same strain.

### Strain typing using the repetitive sequence-based PCR method

Genomic DNA was extracted and purified using an UltraClean Microbial DNA Isolation kit (MO BIO, CA, USA) from the vegetative cells after the cultivation. DNA concentration was measured using a NanoDrop ND-1000 spectrophotometer (Thermo Fisher, MA, USA). Purified DNA was diluted to 25–50 ng/μL with ultrapure water. Rep-PCR was performed using the DiversiLab *Bacillus* kit (SYSMEX bioMérieux, Tokyo, Japan) according to the manufacturer’s protocol. The PCR products were analyzed using DiversiLab using microfluidics chip technology with an Agilent 2100 Bioanalyzer (Agilent Technologies, Tokyo, Japan). The data from DiversiLab for each strain consisted of a visualized fluorescence intensity corresponding to a band pattern, a dendrogram from an unweighted pair group method with arithmetic mean (UPGMA), and a similarity matrix between each strain. The strains having more than 95% in similarity on the basis of the data from DiversiLab were grouped into the same group. The strains having similarity more than 99% were regarded as an identical strain. Independent experiment was repeated three times.

### Ribotyping

The ribotypes of the strains were determined by using a RiboPrinter™ Microbial Characterization System (DuPont, DE, USA) with the restriction enzyme, *Eco*RI. This automated ribotyping system includes each step, such as bacterial cell lysis, restriction enzyme cleavage, electrophoresis, blotting, and detection [20]. The strains having similarity more than 0.9 were regarded as a same ribogroup.

### Comparison of physiological properties using API 50 CH

The carbohydrate utilization of the bacterial strains was determined by using an API 50CH system (SYSMEX bioMérieux, Tokyo, Japan) according to the manufacturer’s instructions. Color changes were confirmed after incubation for 24 and 48 h at 45°C.

### DNA sequencing of 16S rRNA and *gyrB*

Parts of the 16S rDNA and *gyrB* genes were amplified from the genomic DNAs prepared from each of the bacterial strains by PCR using the primers described previously [21]. Multiple sequence alignment of 16S rRNA and *gyrB* was performed using open source software ClustalX2 V2.1 (http://www.clustal.org/clustal2/), after adjusting the length of nucleotide sequence of each strain with Seaview (http://doua.prabi.fr/software/seaview). Bootstrap values were calculated by the neighbor-joining method [22] using ClustalX2. The phylogenetic tree based on the ClustalX2 analysis was displayed using open-source NJplot software (http://doua.prabi.fr/software/njplot).

### Ribosomal internal transcribed spacer region analysis

Polymorphism analysis of the internal transcribed spacer (ITS) region of the 16S-23S ribosomal DNA sequence was performed according to the method of Daffonchio et al. [23]. The template DNA was prepared from each of the strains using Prepman Ultra (Applied Biosystems, CA, USA). PCR was done with TaKaRa ExTaq Hot Start Version (Takara Bio, Shiga, Japan), template DNA, and the primers in a reaction volume of 50 μL. The PCR conditions were initial denaturation at 94°C for 2 min, then 30 cycles of denaturation at 94°C for 1 min, annealing at 55°C for 1 min, and extension at 72°C for 1 min, and final extension at 72°C for 2 min.

## Results

### Phylogenetic analysis of *B*. *coagulans* strains based on DNA sequence of 16S rRNA and *gyrB*

**Fig 1:**
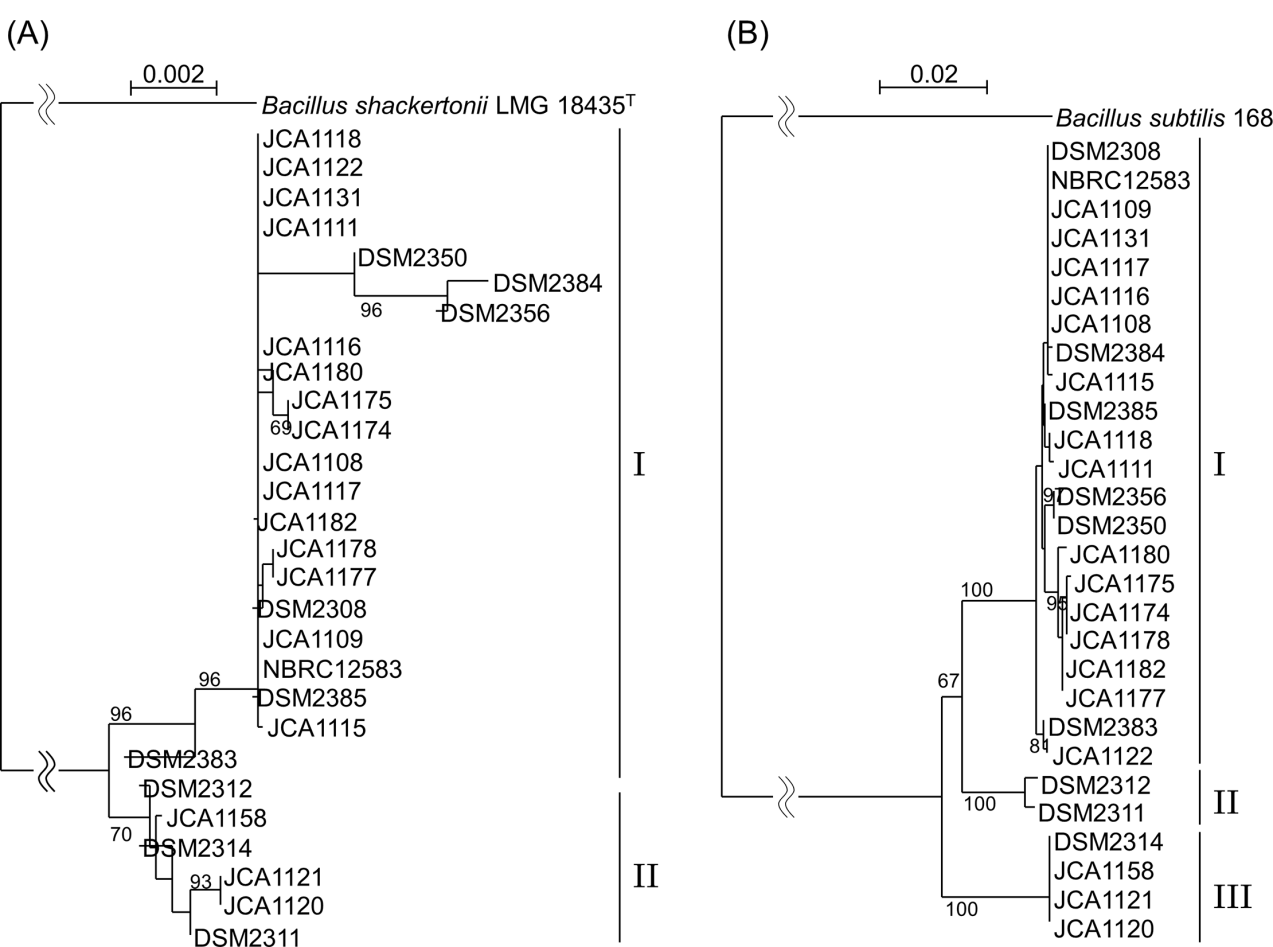
**Phylogenetic trees of *Bacillus coagulans* strains based on nucleotide sequences of (A) 16S rRNA and (B) *gyrB*.** The trees were constructed using the neighbor-joining method, and genetic distances were computed using Kimura’s model. Numbers at the nodes indicate percentages of 1000 bootstrapped trees. The bars indicate genetic distances. *Bacillus subtilis* NBRC13419^T^ and 168 strains were used as the outgroups for phylogenetic analyses based on 16S rRNA and gyrB, respectively. The Roman numbers indicate the different clustering.

Phylogenetic trees derived from the 16S rRNA gene (1458 bp) and *gyrB* (1179 bp) sequences separated into two and three clusters, respectively (Fig 1). Six strains, DSM2311, DSM2312, DSM2314, JCA1120, JCA1121, and JCA1158, were clearly separated from the major cluster for both genes. Other strains were included in major cluster I of both genes. Although *gyrB* gene sequencing has been used phylogenetic study of genus *Bacillus* [21] because its evolutional speed is faster than 16S rRNA gene, it was difficult to distinguish *B*. *coagulans* strains using these housekeeping gene sequence analysis.

### Polymorphism analysis of the internal transcribed spacer region

**Fig 2:**
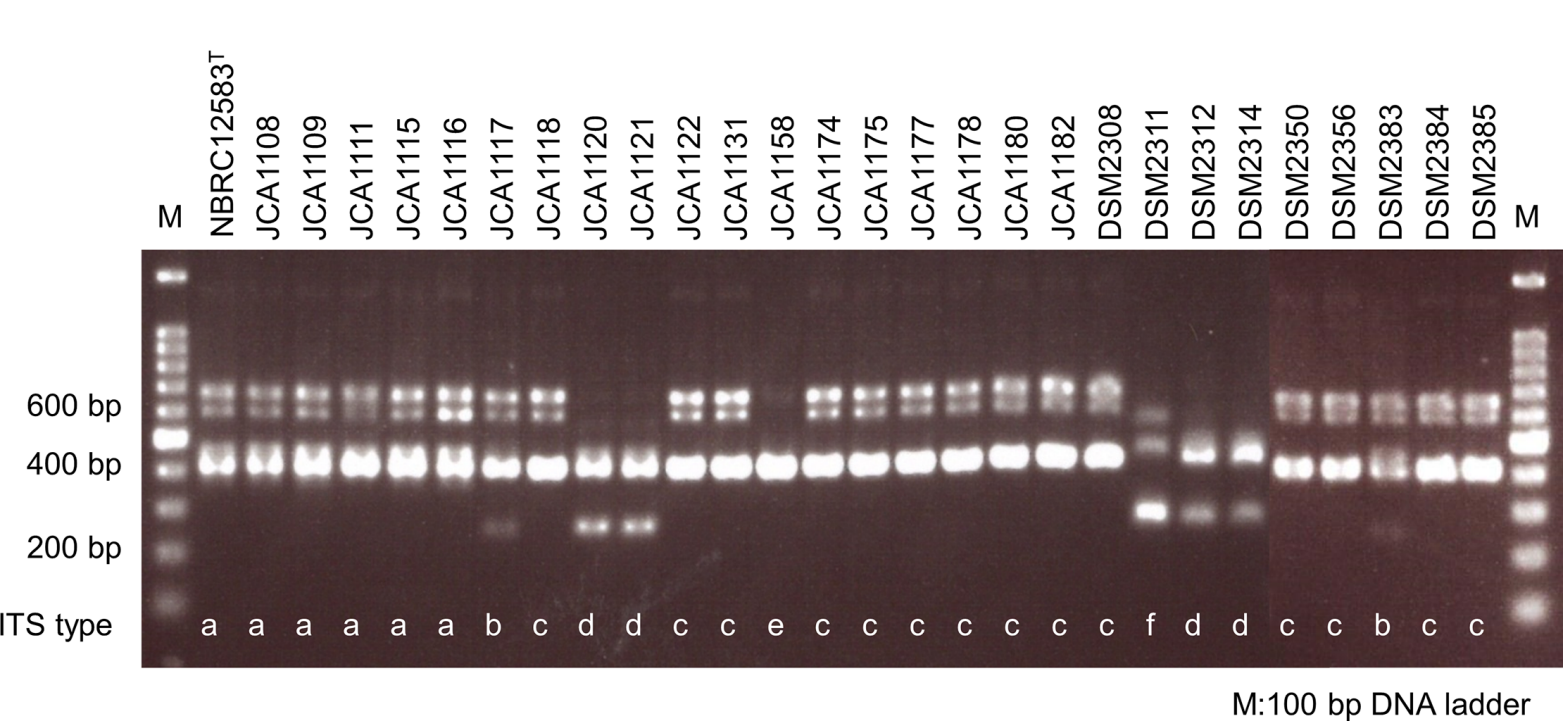
Agarose gel electrophoresis patterns of PCR products showing the internal transcribed spacer (ITS) region in *Bacillus coagulans* strains. The alphabets indicated the each group which separated by the difference of ITS region.

By PCR analysis of the ITS regions, 28 *B*. *coagulans* strains were grouped into 6 groups (Fig 2). The two bands at about 600 and 700 bp existed in the strains included in the major cluster of single locus strain typing of *16S rRNA and gyrB* genes, except for the 6 strains listed above.

### Strain typing using repetitive sequence-based PCR

**Fig 3:**
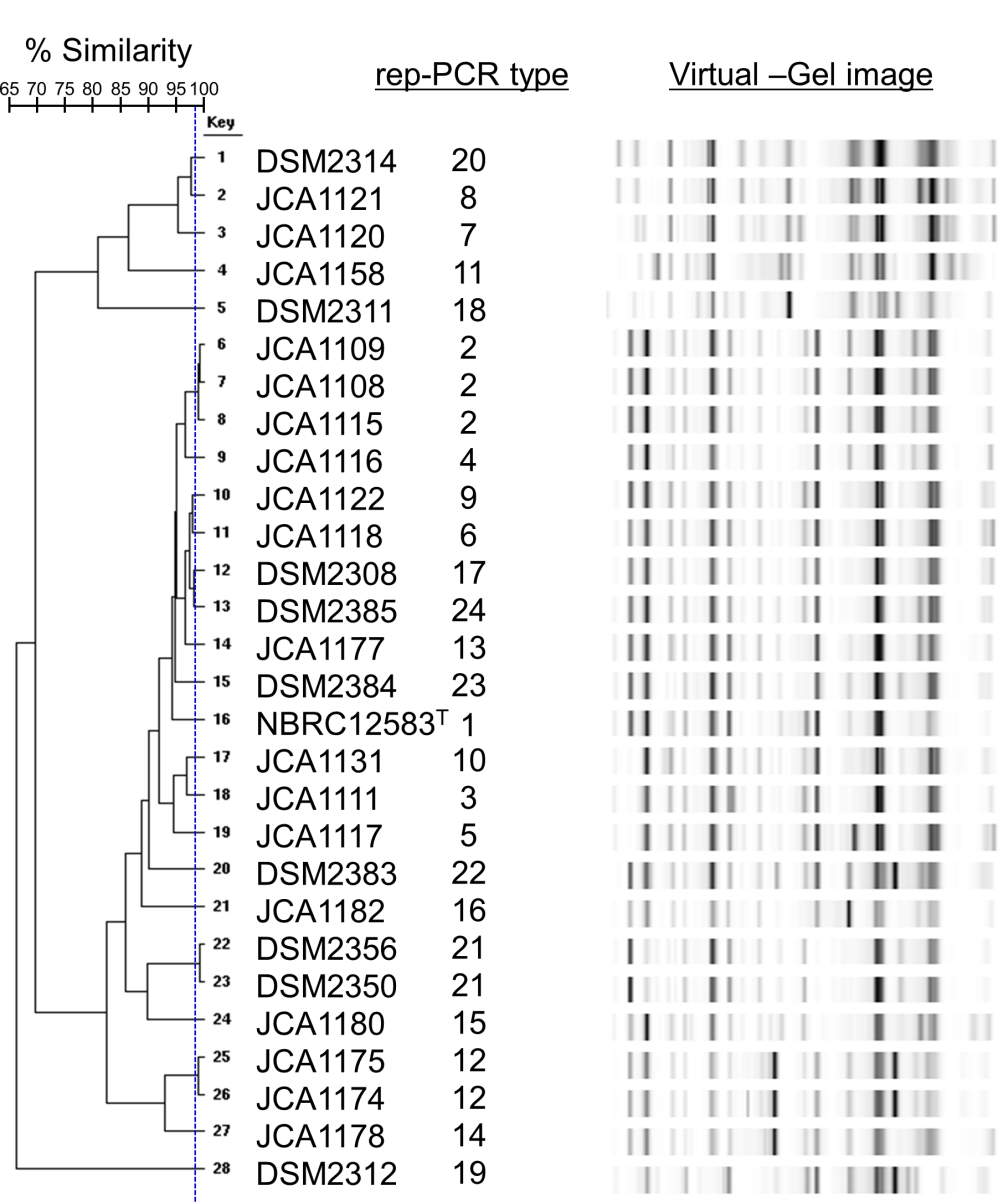
Strain typing of *Bacillus coagulans* based on rep-PCR analysis and band patterns of rep-PCR. The number symbols of rep-PCR type indicated the each group which separated by rep-PCR analysis.

Twenty-eight strains of *B*. *coagulans* were discriminated into 24 groups according to rep-PCR type by rep-PCR analysis (Fig 3). Three groups of rep-PCR type: rep-PCR type No. 2 (JCA1108, JCA1109, and JCA1115); rep-PCR type No. 12 (JCA1174 and JCA1175); and rep-PCR type No. 21 (DSM2350 and DSM2356) showed very high similarity (≥ 99%).

### Microbiological analysis by matrix-assisted laser desorption ionization time-of-flight mass spectrometry

**Fig 4:**
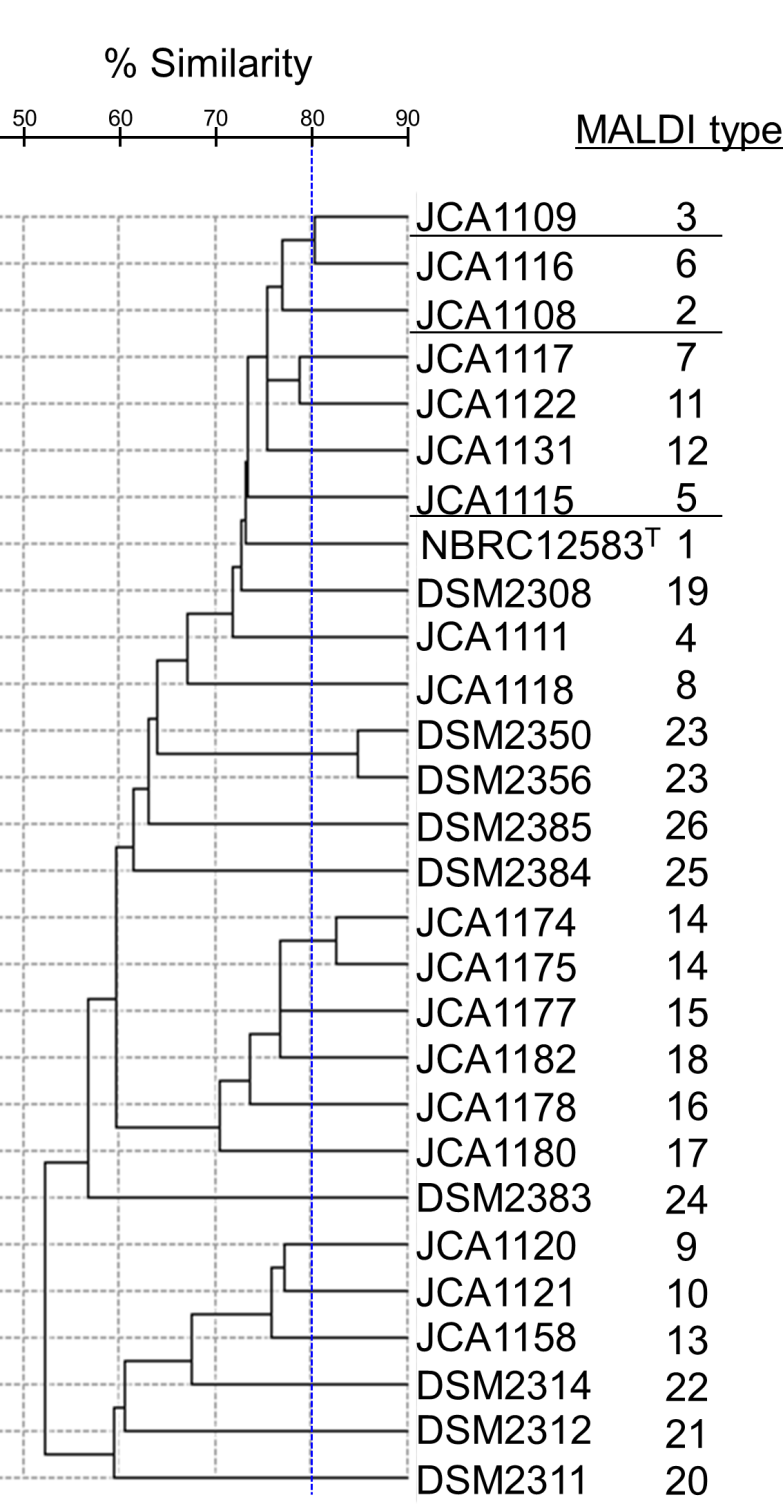
Dendrogram of *Bacillus coagulans* MALDI-TOF MS spectra based on SARAMIS inner species relative cluster analysis. Separation of MALDI type was compared with repetitive-PCR (rep-PCR) type.

The 28 strains of *B*. *coagulans* were discriminated into 26 groups of MALDI type based on the similarity of MALDI-TOF MS patterns (Fig 4). The strains included in 2 groups of MALDI type, MALDI type No. 14 (JCA1174 and JCA1175), and MALDI type No. 23 (DSM2350 and DSM2356) were suggested to be identical by the analysis using SARAMIS software (Similarity: ≥ 80%). The 3 strains of rep-PCR type No. 2 (JCA1108, JCA1109, and JCA1115) were separated into different MALDI types (Fig 4, underlined).

### Ribotyping

RiboPrinter system with *Eco*RI separated the 28 strains of *B*. *coagulans* into 23 ribogroups including four ribogroups: JCA1108, JCA1115, and JCA1116 (Ribogroup No. 2); JCA1121 and DSM2314 (Ribogroup No. 8); JCA1174 and JCA1175 (Ribogroup No. 12); DSM2350 and DSM2356 (Ribogroup No. 20) (Table 2) and 19 individual ribotypes (Table 2).

**Table 2:** Summary of grouping of *B*. *coagulans* strains by the different typing methods.

Strain	Ribogroup	rep-PCR type	MALDI type	Carbohydrate utilization	ITS type
**NBRC12583**	1	1	1	1	a
**JCA1108**	2	2	2	2	a
**JCA1109**	3	2	3	3	a
**JCA1111**	4	3	4	4	a
**JCA1115**	2	2	5	5	a
**JCA1116**	2	4	6	6	a
**JCA1117**	5	5	7	7	b
**JCA1118**	6	6	8	8	c
**JCA1120**	7	7	9	9	d
**JCA1121**	8	8	10	10	d
**JCA1122**	9	9	11	11	c
**JCA1131**	10	10	12	12	c
**JCA1158**	11	11	13	13	e
**JCA1174**	12	12	14	14	c
**JCA1175**	12	12	14	14	c
**JCA1177**	13	13	15	15	c
**JCA1178**	14	14	16	16	c
**JCA1180**	15	15	17	17	c
**JCA1182**	16	16	18	18	c
**DSM2308**	17	17	19	19	c
**DSM2311**	18	18	20	20	f
**DSM2312**	19	19	21	21	d
**DSM2314**	8	20	22	22	d
**DSM2350**	20	21	23	23	c
**DSM2356**	20	21	23	23	c
**DSM2383**	21	22	24	24	b
**DSM2384**	22	23	25	25	c
**DSM2385**	23	24	26	26	c

### Carbohydrate utilization of various *B*. *coagulans* strains

**Fig 5:**
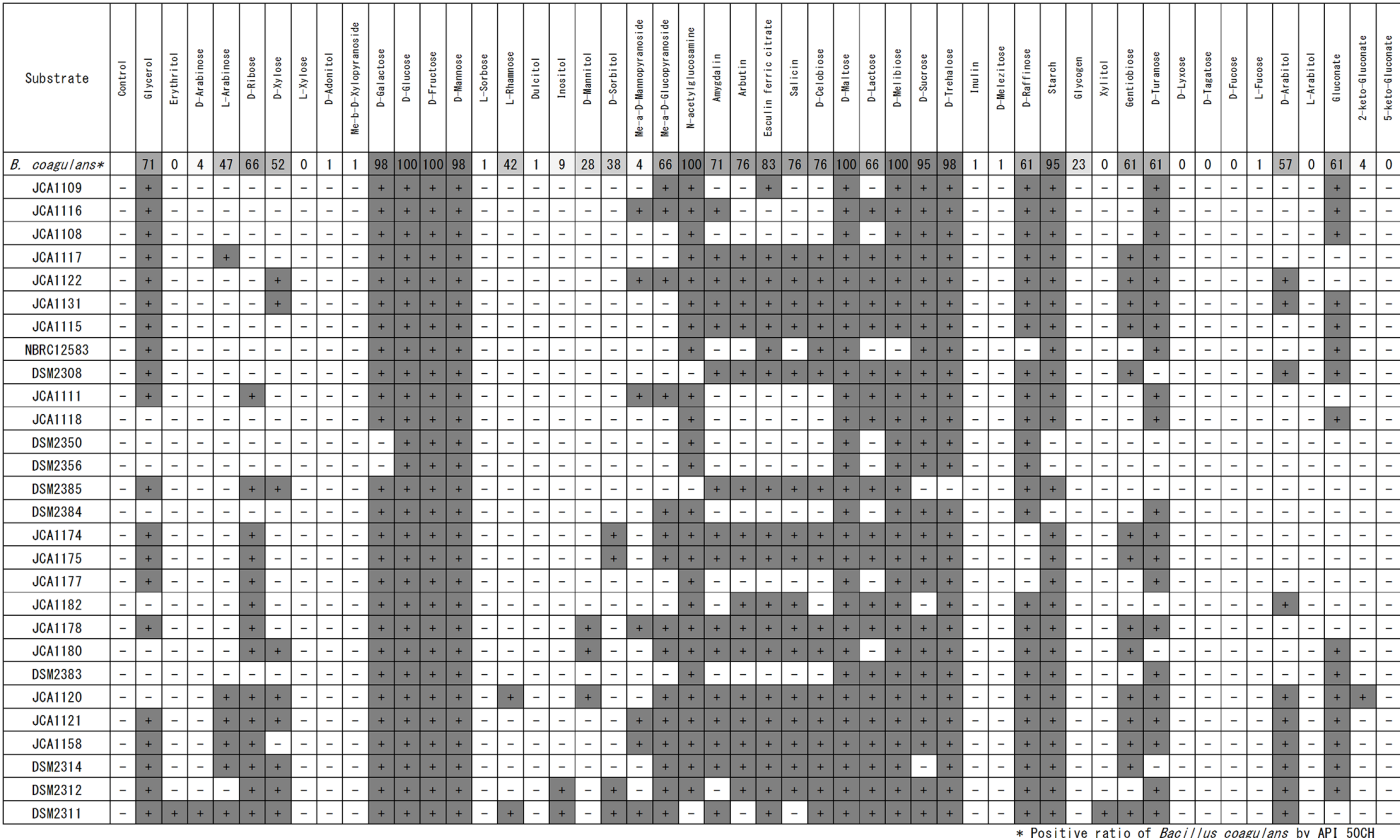
Carbohydrate utilization by various *Bacillus coagulans* strains. Gray and white boxes indicate positive and negative, respectively.

The 28 strains of *B*. *coagulans* possessed various types of carbohydrate utilization (Fig 5). The utilization of glycosides such as amygdalin, arbutin, esculin, salicin, and D-celobiose was largely different between the strains. Although 4 strains, JCA1108, JCA1109, JCA1115, and JCA1116, that exhibited strain similarity in the rep-PCR and/or Ribotyping analyses, also showed similar utilization of carbohydrates, the metabolic abilities of the glycosides were slightly different. On the other hand, JCA1174 and JCA1175 showed the same carbohydrate utilization. The same was true on DSM2350 and DSM2356. The strains JCA1174 and JCA1175 could not utilize D-galactose and starch, whereas the strains DSM2350 and DSM2356 were able to utilize these sources. The latter two strains utilized D-sorbitol, but not D-raffinose.

## Discussion

The discriminatory power of strain typing of *B*. *coagulans* was compared among rep-PCR, MALDI-TOF MS, ribotyping, and carbohydrate utilization in the point of view of the precision and rapidity. Firstly, single gene sequencing analysis of the housekeeping genes 16S rDNA and *gyrB* provided only limited information for identification of species. It was difficult to distinguish *B*. *coagulans* strains by DNA sequencing of housekeeping genes and the PCR band patterns of ribosomal ITS region because of their small difference in DNA sequence. MLST might be useful for strain typing of *B*. *coagulans* because the genes that having higher evolution rates than housekeeping genes are usually chosen as the targets for MLST analysis [24]. MLST has been used as the standard method for a food-borne pathogen, *Bacillus cereus* [25]. MLST allows the characterization of the genetic diversity between environmental isolates using international databases and shows good reproducibility on the bacterial species with long-term epidemiological studies. However, it is difficult to apply to discriminate of bacteria including *B*. *coagulans* with poor genomic information in the databases and unidentified isolates from the environment. PFGE, the other gold standard of a strain typing method, needs the technical experience and is time-consuming. In this study, the rep-PCR and MALDI-TOF MS methods showed approximately equal performance in strain typing compared with Ribotyping, one of the most common and easy reference typing methods in the present circumstances [26]. Rep-PCR analysis targets repetitive sequences that are widely distributed in prokaryotic genomes. MALDI-TOF MS targets proteins with the low-molecular-weight ranges of *m/z* 3000 to 20000, especially ribosomal and envelope proteins. These analytical targets might contribute to the high discriminatory power of strain typing. We found that two groups, JCA1174 and JCA1175, and DSM2350 and DSM2356, showed high similarity by both rep-PCR and MALDI-TOF MS. In addition, carbohydrate utilization was fully consistent between DSM2350 and DSM2356 and between JCA1174 and JCA1175. The profile of carbohydrate utilization is one of the most important phenotype for the discrimination of bacterial strains [27, 28]. These results suggested that the bacterial protein profiles by MALDI-TOF MS are highly correlated with sequence-based strain typing methods as well as species identification. A recent study demonstrated the MALDI-TOF MS method was similar to MLST and superior to RAPD analyses in accuracy of strain typing of *Klebsiella pneumoniae*, which is an important pathogen in nosocomial infections [29]. In contrast, *Berrazeg et al*. reported that there was no direct correlation between MALDI-TOF MS-based typing and MLST for *K*. *pneumoniae* after consideration of different experimental conditions and analytical algorithms [30]. In our experimental conditions with different MALDI-TOF algorithms, the strain typing results of *B*. *coagulans* were slightly different from our present study (data not shown). The appropriate analysis algorithm and cut-off settings of mass spectra are important for the development of strain typing by MALDI-TOF MS.

Strain typing using mass spectra by MALDI-TOF MS has already been proposed in the S10-GERMS (S10-spc-alpha operon gene encoded ribosomal protein mass spectrum) method [31–34]. The S10-GERMS method is focused on the S10-spc-alpha operon, where at least half of all ribosomal proteins are encoded. Some mass spectra can be correlated with theoretically calculated *m/z* ion peaks of ribosomal subunit proteins that are species- or strain-specific. This method is useful for accurate strain typing because of the quantitative correlation between the experimentally observed *m/z* values and theoretical values. However, it is difficult to apply sequence information of the S10-spc-alpha operon with unknown isolates or unidentified species from a standpoint of rapidity and versatility.

Our results suggested that the typing in combination of MALDI-TOF MS and rep-PCR analyses was effective for the strain typing of *B*. *coagulans*. The target proteins of MALDI-TOF MS, including ribosomal and envelope proteins, and repetitive sequences targeted by rep-PCR in the bacterial genome were highly related. Consequently, it was possible to achieve precise discrimination between different strains of *B*. *coagulans*. Further investigations on various strains with different characteristics among the same bacterial species are needed to demonstrate the usability and versatility of the MALDI-TOF MS-based method as a rapid bacterial strain typing method.
